# The effect of esketamine on perioperative neurocognitive dysfunction in elderly patients undergoing gastrointestinal tumor surgery: a randomized double-blind controlled study

**DOI:** 10.3389/fmed.2025.1653132

**Published:** 2025-10-06

**Authors:** Xiaoyan Ma, Jiarun Qin, Min Li, Yifan Liu, Wenli Yu

**Affiliations:** ^1^Department of Anesthesiology, Changzhi People‘s Hospital, Changzhi, China; ^2^The First Central Clinical School, Tianjin Medical University, Tianjin, China; ^3^Department of Anesthesiology, Changzhi People‘s Hospital Affiliated to Changzhi Medical College, Changzhi, China; ^4^Department of Anesthesiology, Tianjin First Center Hospital, Tianjin, China

**Keywords:** esketamine, gastrointestinal tumor operation, elderly patient, PND, neuroinflammation

## Abstract

**Background:**

We aimed to whether esketamine induction and maintenance of general anesthesia could reduce the incidence of perioperative neurocognitive dysfunction (PND) in elderly patients undergoing gastrointestinal tumor surgery and explore the related mechanisms preliminarily.

**Patients and methods:**

A total of 153 elderly patients were divided into two groups: a control group (group C, *n* = 75) and an esketamine group (group K, *n* = 78). In group K, 0.3 mg/kg esketamine was injected intravenously during anesthesia induction, and 0.3 mg·kg^−1^·h^−1^ was injected intravenously to maintain anesthesia. In group C, esketamine was replaced with an equal volume of normal saline. The Pittsburgh Sleep Quality Index (PSQI) was used to evaluate sleep quality 1 day before surgery and at 1, 3, 7, and 30 days after surgery. A battery of neurological tests was used to assess cognitive function 1 day before surgery and 7 and 30 days after surgery. Serum IL-6, TNF-α, NSE and Aβ1–42 concentrations were tested by enzyme-linked immunosorbent assay before surgery, at the end of surgery and 1 day after surgery.

**Results:**

The incidence of PND in group K at 7 days after surgery was lower than that in group C (*P* < 0.05). Compared with that in group C, the PSQI score in group K was lower at 1 and 3 days after surgery (*P* < 0.05). Compared with those in group C, the TNF-ɑ concentration in group K were lower both after surgery and 1 day after surgery (*P* < 0.05), and the IL-6, NSE and Aβ1-42 concentration were lower at 1 day after surgery (*P* < 0.05).

**Conclusion:**

The use of esketamine for anesthesia induction and maintenance in elderly patients undergoing gastrointestinal tumor surgery inhibited inflammation, alleviated neuronal injury and degeneration, improved postoperative sleep quality and cognitive function, and reduced the incidence of PND.

## Introduction

Perioperative neurocognitive dysfunction (PND) is characterized by abnormal phenomena such as inattention, memory loss, learning decline and mood swings (1). PND is a major postoperative neurological complication in elderly patients, which prolongs hospital stays, increases medical expenses, increases disability and mortality rates, and places a heavy burden on families and society (2–5). Due to the decline of various organ functions and a variety of systemic diseases before surgery, elderly patients' tolerance to surgery and anesthesia trauma is reduced, and the incidence of PND after surgery is significantly increased (6). Despite the improvements in surgical techniques and anesthesia management, the incidence of PND in postoperative elderly patients is still 10%−54% (7).

Abnormal secretion of central neurotransmitters (8), the inflammatory response induced by surgical trauma (9), vascular endothelial dysfunction and blood–brain barrier dysfunction (10, 11) are important pathophysiological mechanisms of postoperative cognitive impairment. Gastrointestinal surgery can not only cause mechanical injury to gastrointestinal tissue but also cause changes in the intestinal flora and increase intestinal mucosal permeability. A large number of inflammatory factors, injury-related molecular patterns, metabolites and neurotoxic factors enter the nervous system through the gut-brain axis, causing functional and behavioral abnormalities in the central nervous system.

Esketamine is an NMDA receptor antagonist, which has sedative and analgesic effects, and can alleviate the hyperalgesia caused by remifentanil (12). While esketamine is generally well-tolerated at the doses used in clinical practice, it is important to note that higher doses may be associated with potential side effects such as dissociation and hallucinations (13).

Esketamine can enhance the synaptic plasticity of human hippocampal neurons (14), increase immune activity, inhibit the inflammatory response, and improve cognitive function (15). A recent study of the human metabolome showed that esketamine can reduce the content of branched-chain amino acids in circulation, promote the synthesis and release of noradrenaline in the brain, and improve patients' depression and cognitive function (16). There are limited reports on the correlation between esketamine and perioperative neurocognitive impairment in elderly patients undergoing gastrointestinal surgery. This study aimed to investigate the effects of esketamine on perioperative neurocognitive impairment and related factors in elderly patients undergoing gastrointestinal surgery.

## Materials and methods

### Patients and study protocol

The study was approved by the Ethics Committee of Changzhi People's Hospital (2021K60) and registered in the Chinese Clinical Trial Registry (ChiCTR2200064076). Before the study, patients selected for gastrointestinal tumor resection under general anesthesia from October 2022 to October 2023 were informed of the study protocol and signed informed consent. Inclusion criteria: age ≥65 years old; ASA I–III grade; BMI 18–30 kg/m^2^; NYHA I–II grade; no serious hepatic and renal dysfunction. Exclusion criteria: having a mental illness or taking antipsychotic drugs; allergic to esketamine; severe abnormal liver and kidney function (severe abnormal liver function: ALT, AST, bound bilirubin, total bilirubin, one of the values is >2 times the upper limit of normal; severe renal dysfunction: Cr clearance < 30 ml/min); patients with unstable angina pectoris or myocardial infarction within 3 months; preoperative blood pressure ≥180/110 mmHg; hyperthyroidism; patients with high intraocular pressure.

### Randomization, blinding, and data collection

The sequence number was obtained according to the random number table method, and a random card was used to seal the samples in the envelope. The envelope was opened after the patient entered the operating room. In this study, according to the random cards, the subjects were divided into a control group (group C) and an esketamine group (group K), and the allocation ratio was 1:1. A nurse anesthetist prepared the medication, released the envelopes and coordinated the information between the researchers. Study subjects, family members, data collection, postoperative follow-up and neurological scale testers were not clear about the grouping. The data statisticians were blinded to the research plan, which is significance to ensure the truth of data collation and analysis. The anesthesia management staff consisted of one senior anesthesiologist and three attending anesthesiologists. The senior anesthesiologist was responsible for anesthesia induction and maintenance. The attending anesthesiologist was responsible for the data collection and other work. The researchers did not know each other's results during the study.

### Anesthesia protocol

The patients fasted for 6 h and provided free access to water for 2 h before surgery. After entering the operating room, all patients were administered standard monitoring, including ECG, oxygen saturation, non-invasive blood pressure, body temperature and the Bispectral Index (BIS). Invasive arterial pressure was measured via radial artery puncture, and central venous pressure was measured via right internal jugular vein puncture. Anesthesia was induced by midazolam [(0.03 mg/kg), 2 ml/10 mg, Jiangsu Enhua Pharmaceutical Co., Xuzhou, China], propofol [(1–2 mg/kg), 0.2 g/20 ml, Beijing Fresenius Kabi Pharmaceutical Co., Beijing, China], and esketamine [(0.3 mg/kg), 50 mg/2 ml, Jiangsu Hengrui Pharmaceutical Co., Lianyungang, China], sufentanil [(0.2–0.4 μg/kg), 1 ml/50 μg, Yichang Renfu Pharmaceutical Co., Yichang, China], and cisatracurium [(0.2 mg/kg), 10 mg/5 ml, Nanjing Jianfa Chemical Pharmaceutical Co., Nanjing, China]. After the BIS decreased to < 55 and the muscle completely relaxed, a tracheal catheter was inserted under the visual laryngoscope and connected to the anesthesia machine. All patients were mechanically ventilated with 60% oxygen at a 2 L/min flow rate, a tidal volume of 6–8 ml/kg, a frequency of 12–15 breaths/min, and an end-expiratory carbon dioxide (PETCO2) concentration of 35–45 mmHg. Anesthesia was maintained by injecting 0.05–0.15 μg.kg^−1^·min^−1^ remifentanil, 2–4 mg·kg^−1^·h^−1^ propofol, 0.1 mg·kg^−1^·h^−1^ cisatracurium, 1%−2% sevoflurane, and 0.3 mg·kg^−1^·h^−1^ esketamine in group K. Group C was given an equal volume of normal saline. During the operation, all patients had an SVV < 13%, a MAP fluctuation range < 10% before the operation, a BIS value 40–60, and a body temperature above 36 °C. The patients stopped injecting esketamine 1 h before the end of surgery. The inhalation of sevoflurane and infusion of cis-atracurium were stopped 30 min before the end of surgery. At the end of the operation, remifentanil and propofol were discontinued with a connecting analgesic pump. Postoperative analgesia was achieved using a multimodal approach. All patients received a patient-controlled analgesia (PCA) pump with the following components: sufentanil [(2 μg/kg), 1 mL/50 μg, Yichang Renfu Pharmaceutical Co., Yichang, China], nalbuphine injection [(30 mg), 10 mg/2 ml, Beijing TaiDe Pharmaceutical Co., Beijing, China], and palonosetron [(0.25 mg), 0.25 mg/5 ml, Hangzhou Jiuyuan Gene Engineering Co., Beijing, China], diluted to 100 ml with normal saline. The PCA pump was set with a maintenance dose of 2 ml/h and a bolus dose of 0.5 ml, with a lockout time of 15 min. After the operation, the patient was transferred to the PACU with a tracheal catheter.

## Observation indices and observation methods

### Baseline data

Baseline data, including age, sex, weight, height, previous history (hypertension, diabetes, cerebrovascular disease) and years of education, were recorded for the two groups.

### Main outcome

The main outcome of this study was the incidence of PND within 30 days after surgery in both groups. According to the age of receiving education and the condition of the hospital cognitive center, the cognitive function of the elderly patients was evaluated by using the electronic neuropsychological composite scale from the aspects of attention, memory, executive ability and language ability. The combined scale included the Mini-Mental State Examination (MMSE), Digital Span Test (DST), Color Trail Test (CTT), Symbol Digit Modalites (SDWT) and Verbal Fluency Test (VFT). The cognitive function of the enrolled patients was evaluated by professional testers at the cognitive center 1 day before surgery, 7 days after surgery, and 30 days after surgery. Healthy elderly individuals older than 65 years were included in the physical examination at the cooperative community hospital of our hospital and were tested with a combination of neuropsychological tests three times at intervals of 7 days and 30 days as the cognitive function assessment control group. The “Z score method” (17) was used to diagnose PND. The specific method is to record the control group as group S, the baseline score of the scale and the score of the interval time as N0 and N1, respectively, the mean difference of the two scales in group S as ΔN, the standard deviation as ΔSD, and the two scale values of the researcher as A and B, respectively, then the *Z* value = [Δ*N*– (*B*–*A*)] ÷ ΔSD. When the *Z* value of at least two subgroups of cognitive function scale scores was ≥2, the patient was diagnosed with PND.

### Secondary outcome

1. Sleep quality was assessed using the Pittsburgh Sleep Quality Index (PSQI) 1 day before surgery and 1, 3, 7, and 30 days after surgery. (2) Intraoperative data, including intraoperative blood loss, operation type, operation time, sufentanil dosage, blood transfusion volume and fluid volume, were recorded. (3) Serum indices were detected at three time points: before surgery (T0), after surgery (T1) and 1 day after surgery (T2). The serum concentrations of tumor necrosis factor alpha (TNF-ɑ), interleukin-6 (IL-6), neuron-specific enolase (NSE) and amyloid β1-42 (Aβ1-42) were detected via ELISA.

### Sample size and statistical analysis

This was a randomized controlled trial, and the main outcome was the incidence of PND. The sample size was calculated using PASS15.0 software. In this study, α = 0.05 and β = 0.1 on the two-tailed test were used, and the results of the pretest study showed that the incidence of PND at 7 days after surgery was 28.71% in group C and 11.32% in group K. The sample size with statistically significant differences between groups C and K was calculated to be 71 patients, with a 20% missing sample rate and a 25% incidence of postoperative delirium; thus, 120 patients were included in each group, for a total of 240 patients in the two groups.

SPSS 23.0 statistical software was used for statistical analysis. Measurement data that conformed to a normal distribution were represented as $\bar{x}\pm s$ and were compared by “*t*” tests. Non-normally distributed data were presented as medians (quartiles) [M(Q1, Q3)] and were compared using the rank-sum test (Mann–Whitney *U* test). Count data were expressed as the number of patients or percentage and were compared by the chi-square test or Fisher's exact probability test. Single factor analysis of repeated measures variance was used for the data from repeated measurements with a normal distribution.

## Results

**Figure 1:**
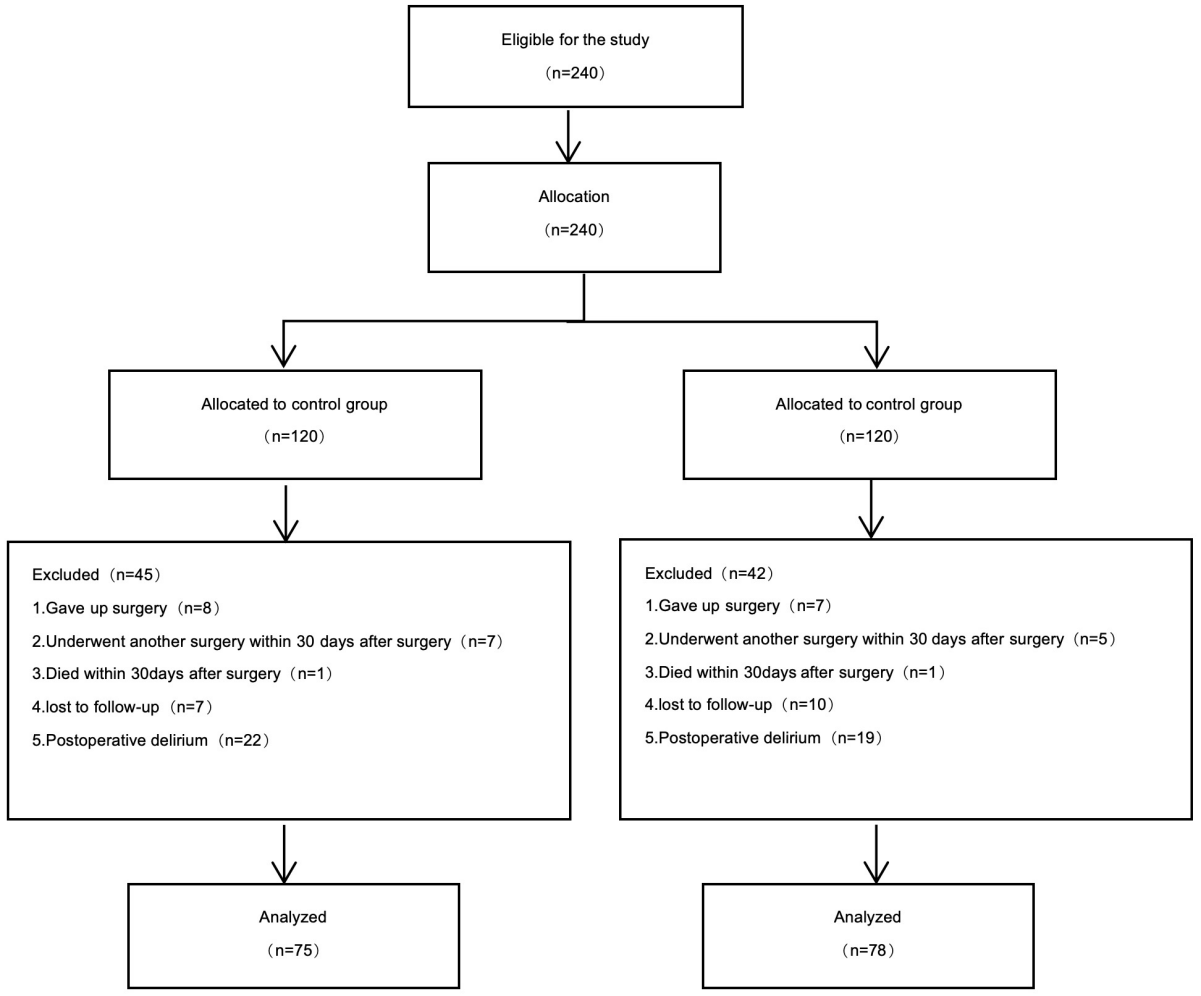
Flow diagram of study participants.

Among the 240 subjects included, 15 patients gave up surgical treatment due to tumor metastasis, 12 patients underwent another surgery within 30 days after surgery, 2 patients died within 30 days after surgery, 17 patients were lost to follow-up to the postoperative scale, and 41 patients suffered from postoperative delirium. Overall, 153 patients were included in the data analysis; 75 patients were in group C, and 78 patients were in group K (Figure 1).

There were no significant differences between the two groups in terms of basic data, such as age, sex, weight, past history or years of education (*P* > 0.05) (Table 1).

**Table 1:** Baseline biological parameters.

**Characteristic**	**C group (*n* = 75)**	***K* group (*n* = 78)**	***t*/*z*/χ^2^**	***P*值**
Age (years)	70 (67, 76)	70 (68, 75)	−0.05	0.963
Sex (*n*, female/male)	25/50	35/43	2.14	0.144
Type of operation (*n*, stomach/intestine)	44/31	56/22	2.91	0.088
Height (cm)	165 ± 7	167 ± 7	−0.70	0.488
Weight (kg)	65 ± 11	67 ± 6	−0.59	0.562
**Past history**				
Hypertension (*n*, %)	14 (40%)	17 (48.57%)	0.52	0.47
Diabetes (*n*, %)	7 (20%)	5 (14.29%)	0.40	0.53
Cerebrovascular disease (*n*, %)	4 (11.43%)	6 (17.14%)	0.47	0.50
Years of education [years, M (Q1, Q3)]	6 (6, 9)	6 (6, 9)	−0.09	0.93

The incidence of PND at 7 days after surgery was significantly lower in group K (8.97%) than in group C (25.33%) (*P* < 0.01). Moreover, there was no significant difference between the two groups in terms of the incidence of PND at 30 days after surgery (*P* > 0.05) (Table 2).

**Table 2:** The incidence of PND (*n*, %).

**Groups**	**7 days after surgery**	**30 days after surgery**
C group (*n* = 75)	19 (25.33%)	8 (10.67%)
K group (*n* = 78)	7 (8.97%)^a^	5 (6.41%)
χ^2^值	7.25	0.89
*P*值	0.007	0.345

^a^*P* < 0.05 vs. C group.

A total of 56 elderly healthy persons were enrolled in community hospitals. There were no significant differences in MMSE, DST, SDMT, VFT or CTT scores among the three neuropsychological combination scale assessments at 7 d or 30 d intervals (*P* > 0.05) (Table 3).

**Table 3:** Community patient neuropsychological combination scale score (*n* = 56, $\bar{x}\pm s$).

**Scale**	**1st day (point)**	**7th day (point)**	**30th day (point)**	***F*值**	***P*值**
MMSE	26.1 ± 1.6	26.3 ± 1.7	26.3 ± 1.8	0.09	0.916
DST	7.7 ± 1.3	7.9 ± 1.3	7.9 ± 1.2	0.21	0.813
SDMT	33.7 ± 3.5	33.9 ± 2.7	34.2 ± 2.3	0.20	0.816
VFT	23.4 ± 3.2	23.0 ± 3.5	23.3 ± 2.9	0.11	0.896
CTT	43.3 ± 6.0	42.1 ± 3.9	43.2 ± 5.6	0.51	0.604

The MMSE, DST, SDMT and VFT scores were lower at 7 days after surgery than at 1 day before surgery, and the CTT scores were greater (*P* < 0.05). There was no significant difference in scale scores at 1 day before surgery between the two groups and the community patient group (*P* > 0.05). The MMSE, DST and SDMT scores in group K at 7 days after surgery were greater than those in group C, and the CTT score decreased (*P* < 0.05) (Table 4).

**Table 4:** Neuropsychological combination scale score (point, $\bar{x}\pm s$).

**Scale**	**C group(n** = **75)**			**K group(n** = **78)**		
	**1 d before surgery**	**7 d after surgery**	**30 d after surgery**	**1 d before surgery**	**7 d after surgery**	**30 d after surgery**
MMSE	26.2 ± 1.4	22.3 ± 1.6^a^	25.5 ± 1.2	26.0 ± 1.1	23.4 ± 1.0^ab^	25.7 ± 1.1
DST	7.5 ± 1.5	5.1 ± 1.3^a^	6.3 ± 1.0	7.9 ± 1.2	6.2 ± 1.5^ab^	6.7 ± 1.5
SDWT	33.4 ± 4.3	25.1 ± 4.3^a^	31.2 ± 2.9	33.9 ± 9.9	28.4 ± 3.3^ab^	31.9 ± 3.5
VFT	23.0 ± 2.5	20.1 ± 1.8^a^	21.5 ± 3.0	23.1 ± 2.9	20.2 ± 2.2^a^	21.3 ± 3.0
CTT	45.2 ± 4.0	53.3 ± 6.0^a^	49.2 ± 5.7	42.7 ± 5.1	48.3 ± 4.1^ab^	46.4 ± 6.7

^a^*P* < 0.05 vs. 1 day before surgery.

^b^*P* < 0.05 vs. C group.

The PSQI scores of the two groups were greater at 1 day, 3 days and 7 days after surgery than at 1 day before surgery (*P* < 0.05). The PSQI scores were lower in the K group than in the C group at 1 day and 3 days after surgery (*P* < 0.05) (Table 5).

**Table 5:** PSQI score [point, *M* (*Q1, Q3*)].

**Group**	**1 d before surgery**	**1 d after surgery**	**3 d after surgery**	**7 d after surgery**	**30 d after surgery**
C group (*n* = 75)	4 (3, 5)	13 (12, 13)^b^	11 (11, 12)^b^	9 (8, 10)^b^	5 (4, 6)
K group (*n* = 78)	4 (4, 5)	12 (11, 13)^ab^	10 (10, 12)^ab^	9 (8, 9)^b^	4 (4, 5)
*Z*值	−0.88	−2.49	−1.98	−0.06	−0.50
*P*值	0.381	0.013	0.048	0.956	0.615

^*a*^*P* < 0.05 vs. the C group.

^b^*P*<*0.05* vs. 1 day before surgery.

There were no significant differences in the dose of sufentanil, anesthesia time, operation time, blood loss or infusion volume between the two groups (*P* > 0.05) (Table 6).

**Table 6:** Intraoperative data ($\bar{x}\pm s$).

**Paramater**	**C group**	**K group**	***t*值**	***P*值**
	***n* = 75**	***n* = 78**		
Dose of sufentanil (μg)	50 ± 11	54 ± 13	−0.83	0.416
Anesthesia time (min)	362 ± 83	304 ± 63	1.99	0.092
Operation time (min)	282 ± 63	240 ± 49	1.919	0.067
Amount of bleeding (ml)	126 ± 55	95 ± 38	1.692	0.104
Fluid volume (ml)	2174 ± 677	1754 ± 426	1.893	0.070

**Figure 2:**
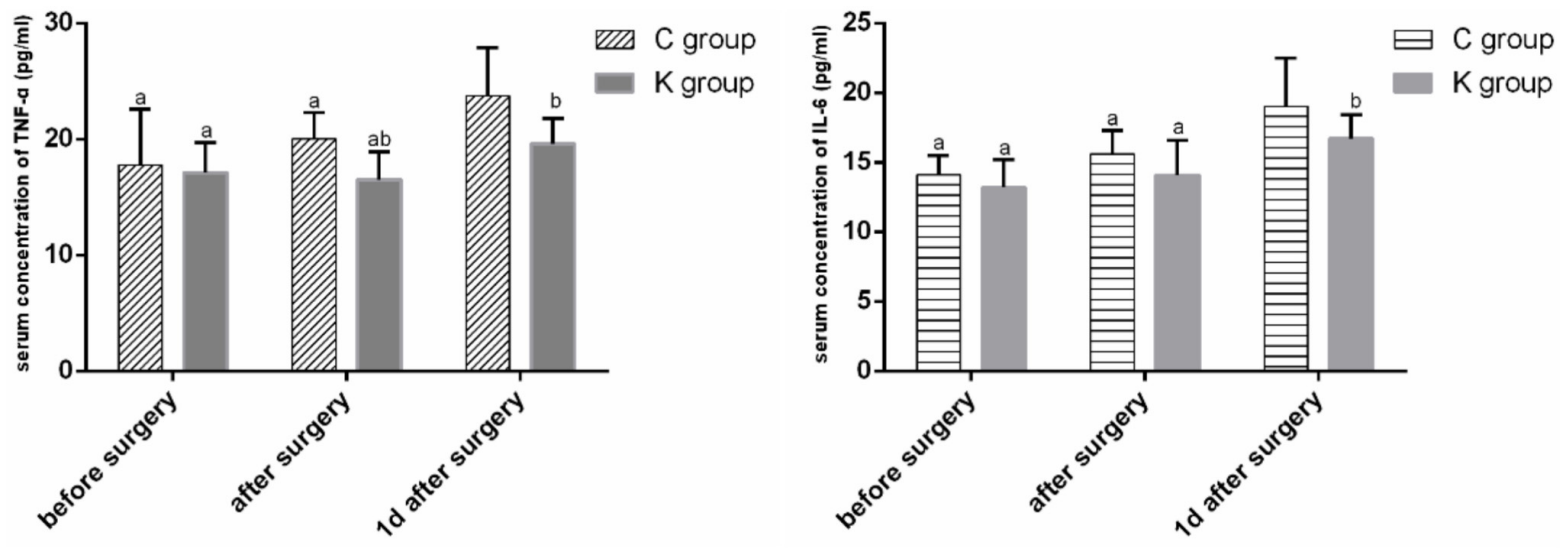
Serum TNF-α and IL-6 concentrations over time. ^a^*P* < 0.05 vs. 1 day after surgery. ^b^*P* < 0.05 vs. C group.

Serum TNF-ɑ and IL-6 concentrations were greater in both groups 1 day after surgery than before and after surgery (*P* < 0.05). The serum TNF-ɑ concentration was lower in group K after surgery and 1 day after surgery than in group C, and the serum IL-6 concentration decreased 1 day after surgery (*P* < 0.05) (Figure 2).

**Figure 3:**
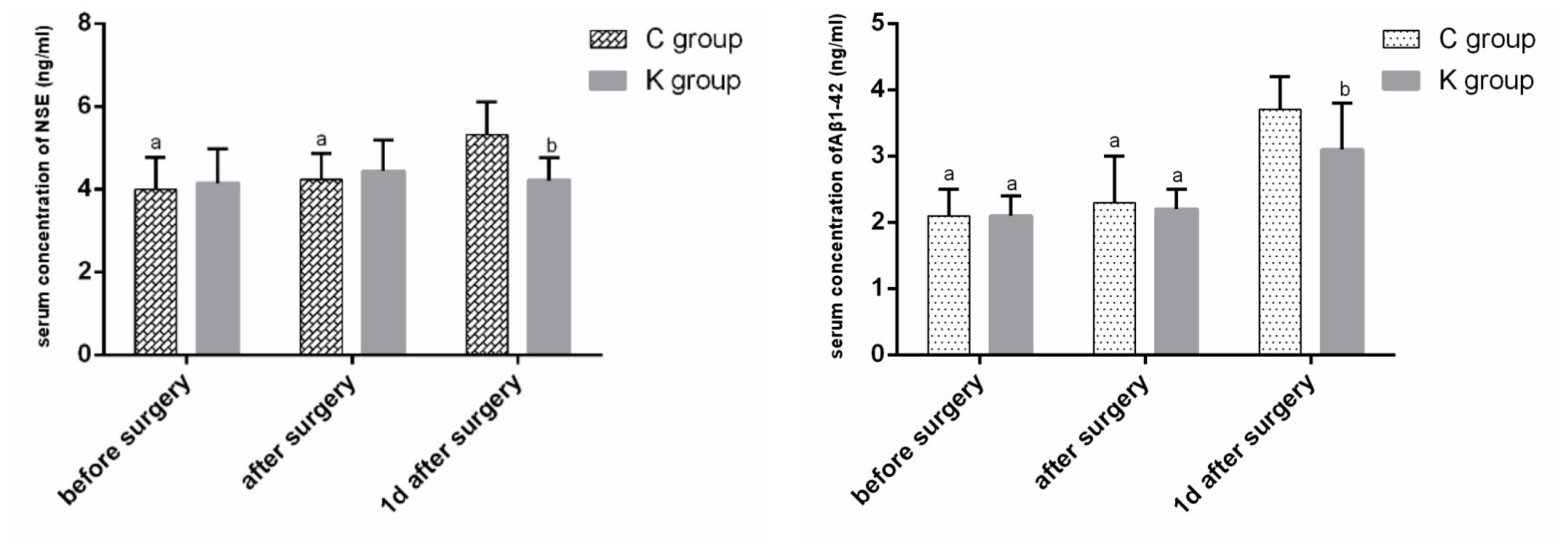
Serum concentrations of NSE and Aβ1-42 over time. ^a^*P* < 0.05 vs. 1 day after surgery. ^b^*P* < 0.05 vs. C group.

The serum concentrations of NSE and Aβ1-42 in group C were greater at 1 day after surgery than before and after surgery (*P* < 0.05). The serum Aβ1-42 concentration in group K was greater at 1 day after surgery than before and after surgery (*P* < 0.05). The serum NSE and Aβ1-42 concentrations were lower at 1 day after surgery in group K than in group C (*P* < 0.05) (Figure 3).

## Discussion

With the rapid development of medical technology, an increasing number of patients have undergone gastrointestinal surgery. PND is a common complication of the postoperative nervous system and has attracted increasing amounts of attention. Approximately 25% of patients experience deterioration of cognitive function after major non-cardiac surgery, and 10% experience persistent cognitive impairment at 3 months. PND can cause long-term cognitive dysfunction if it lasts for 6–12 months, which seriously affects quality of life and places a heavy burden on families and society (18, 19). The “*Z*” score method is an internationally recognized evaluation standard for perioperative neurocognitive disorders (20). Compared to ketamine, esketamine offers a better side effect profile with fewer dissociative and hallucinatory effects. It also has a faster onset and longer duration of action, making it more effective for anesthesia. Additionally, esketamine has less impact on cardiovascular function, which enhances its safety for elderly patients (21). Because higher doses of esketamine may increase the risk of dissociation and hallucinations, we used a judicious dose for induction and maintenance. This strategy provides adequate anesthesia while minimizing such risks, consistent with previous research showing that lower doses reduce the likelihood of severe dissociative effects (22).

In this study, the MMSE, DST and SDMT scores of the two groups were lower at 7 days after surgery than before surgery, and the CTT scores were significantly greater, indicating that the patients had a decrease in cognitive function, attention, memory and executive power at 7 days after surgery. Compared with those in the control group, the MMSE, DST and SDMT scores in the esketamine group were significantly greater, and the CTT scores were significantly lower at 7 days after surgery, indicating that the induction and maintenance of anesthesia injection of esketamine in elderly patients after gastrointestinal surgery can significantly improve the cognitive decline of attention, memory and executive function at 7 days after surgery. Analysis of the “*Z*” score of the composite neuropsychological composite scale revealed that the incidence of PND at 7 days after surgery was significantly lower in group K than in group C. Esketamine improved neurocognitive impairment at 7 days after surgery. Esketamine can improve the plasticity of hippocampal neurons and enhance the function of prefrontal and hippocampal neurons (23). Esketamine can reduce the content of branched-chain amino acids in circulation; promote the synthesis and release of norepinephrine in the brain (16); affect the balance of neurotransmitters; participate in the body's learning, anxiety, mood and other complex functions; and improve cognitive function.

After hospitalization, because of the influence of the environment, noise, disease and pain, patients are prone to sleep disorders during the perioperative period, which can manifest as sleep rhythm disturbance, sleep fragmentation and sleep deprivation. Nighttime sleep plays an important role in learning, memory and maintaining the homeostasis of the brain microenvironment. Sleep is an important physiological process that contributes to the elimination of brain metabolites and the recovery of brain function (24, 25). Postoperative sleep disturbance significantly exacerbates perioperative neurocognitive impairment (26). In the present study, the sleep quality indices 1, 3 and 7 days after surgery were significantly greater than that 1 day before surgery, and sleep quality was significantly lower after surgery. The sleep quality index was significantly lower at 1 d, 3 d and 7 d after surgery in the esketamine group than in the control group, and sleep quality was significantly improved. Active treatment of sleep disorders during the perioperative period can significantly reduce the incidence of PND, and early management of sleep disorders is conducive to improving postoperative cognitive outcomes (27). Postoperative pain is the main factor affecting postoperative sleep. Postoperative pain is inevitable after major surgery, and improper management of pain not only reduces total sleep time (28) but also affects cognitive scores (29). Esketamine has an opioid retention effect, and the postoperative pain relief satisfaction of patients who receive intravenous esketamine significantly increases, the demand for postoperative pain medication significantly decreases, and the quality of rehabilitation significantly improves (30). By acting on NMDA receptors, opioid receptors and cholinergic receptors, esketamine exerts sedative, hypnotic and analgesic effects to improve sleep quality, promote brain function recovery and improve cognitive function. Surgical trauma can cause aseptic inflammatory responses in the body. Released injury-related molecules, such as IL-1, TNF-ɑ, IL-6 and other proinflammatory factors, can trigger a systemic inflammatory response in the body, and improper control of their normal regression can lead to pathological neuroinflammation (31). Peripheral surgical stimulation can activate brain astrocytes and microglia and secrete significant amounts of proinflammatory factors into the brain. Microglia activate and release inflammatory mediators, which is an important pathophysiological mechanism of PND (32). Microglia are activated by binding soluble Aβ through receptors on the membrane surface, where they secrete inflammatory mediators such as TNF-ɑ, IL-6, and IL-1β. These inflammatory mediators activate the complement system, cause inflammation of the central nervous system, affect synaptic connections, and lead to cognitive dysfunction (33). It has been shown that esketamine has significant anti-inflammatory effects in rodents (34, 35). The serum concentrations of the inflammatory factors TNF-ɑ and IL-6 in elderly gastrointestinal patients significantly increased 1 day after surgery. The serum concentration of TNF-ɑ in the esketamine group was significantly lower after surgery and 1 day after surgery than that in the control group, and the serum concentration of IL-6 was significantly lower 1 day after the operation, which indicates that gastrointestinal surgery could cause an increase in the postoperative inflammatory factor TNF-ɑ and in the IL-6 concentration and stimulate the inflammatory response. Esketamine for anesthesia induction and intraoperative maintenance can significantly reduce the postoperative inflammatory response.

Neuron-specific enolase (NSE), which is expressed mainly in neurons and neuroendocrine tissues, is a key enzyme involved in glycolysis and has high sensitivity and specificity for detecting neuronal damage. Surgical trauma stimulates the inflammatory response of the body through the release of a large number of proinflammatory factors, such as IL-1β and IL-6, which causes neuronal injury and destruction of the blood–brain barrier and subsequently leads to neurocognitive disorders. The serum NSE concentration in the control group increased significantly 1 day after surgery, but there was no significant difference in the serum NSE concentration in the esketamine group before, after or 1 day after surgery. The serum NSE concentration was significantly lower in the esketamine group than in the control group 1 day after surgery. Esketamine used in anesthesia induction and maintenance can reduce the serum NSE concentration, reduce neuronal damage, and improve postoperative cognitive function.

Abnormal aggregation and folding of Aβ are major pathological features of degenerative diseases of the central nervous system (36). Aβ has dual dose-dependent effects, both neurotrophic and neurotoxic. Low concentrations of Aβ contribute to the differentiation of immature neurons, and as the concentration increases, Aβ has neurotoxic effects, such as dendrite or axon retraction, and the reduction or disappearance of mature differentiated neuronal cells (37). Aβ can directly or indirectly bind to NMDA receptor subunits at the protrude end and activate NMDA receptors to cause calcium regulation disorders, neuronal death, and synaptic dysfunction (22, 38). Basic and clinical studies have shown that surgery can increase aging-related Aβ protein levels, and a decrease in Aβ protein levels is conducive to improving postoperative cognitive function (39, 40). In the present study, the serum Aβ1-42 concentration in both groups significantly increased 1 day after surgery. The serum Aβ1-42 concentration was significantly lower 1 day after surgery in the esketamine group than in the control group. Gastrointestinal surgery can increase the aging-related serum Aβ1-42 concentration after surgery. The induction and maintenance of anesthesia with esketamine can help reduce the aging-related serum Aβ1-42 concentration and improve postoperative cognitive function. It is speculated that esketamine is an NMDA receptor blocker that can compete with Aβ to block NDMA receptors in the brain, participate in the regulation of synaptic transmission and synaptic plasticity signaling pathways, reverse synaptic damage in the hippocampus, reduce Aβ neurotoxicity, and improve the cognitive function of patients.

## Conclusion

In summary, for elderly patients undergoing gastrointestinal tumor surgery, the administration of esketamine at 0.3 mg/kg for anesthesia induction followed by intravenous infusion at a rate of 0.3 mg·kg^−1^·h^−1^ for anesthesia maintenance can inhibit the inflammatory response, reduce postoperative serum TNF-α and IL-6 concentrations, alleviate neuronal damage and neurodegeneration, decrease serum NSE and Aβ1-42 concentrations, improve sleep quality and cognitive function, and decrease the incidence of PND.

Our research has some limitations. First, it is l single-enter study with a small sample size limited to elderly patients scheduled for gastrointestinal tumor surgery; therefore, the conclusion can not be extended to all elderly surgical patients. Second, because of the time and conditions of the study, neuropsychological combination scale was performed at 7d and 30 d after surgery in this study, which was not extended to 12 months and could not fully evaluated PND. This could contribute to an underestimation of the incidence of PND. Third, the serum index was single, which was lack of specificity. The future study will combine the specific postoperative neurological dysfunction for more thorough research. In summary, a considerable number of evidence supports the use of esketamine for anesthesia induction and maintenance in elderly patients undergoing gastrointestinal tumor surgery can reduce the incidence of PND.

## Data Availability

The raw data supporting the conclusions of this article will be made available by the authors, without undue reservation.
